# Association between cognitive social capital and saliva-related quality of life among head and neck cancer survivors: a cross-sectional study

**DOI:** 10.1007/s00520-026-11361-y

**Published:** 2026-10-22

**Authors:** Alan Carvalho Darós, Jessica Klöckner Knorst, Bruno Emmanuelli, João Alberto de Almeida Gonçalves, Raquel Pippi Antoniazzi, Kívia Linhares Ferrazzo

**Affiliations:** 1 Department of Pathology, Federal University of Santa Maria, Roraima Avenue, 1000, Building 20, Santa Maria, Rio Grande Do Sul 97105-900 Brazil; 2 Department of Stomatology, Federal University of Santa Maria, Santa Maria, Rio Grande Do Sul Brazil

**Keywords:** Head and neck neoplasms, Quality of life, Social capital, Xerostomia

## Abstract

**Purpose:**

To evaluate the association between cognitive social capital and patient-reported saliva-related quality of life among head and neck cancer survivors.

**Methods:**

This cross-sectional study included adults treated with curative intent for squamous cell carcinoma of the lip/oral cavity, oropharynx, hypopharynx, or larynx at a referral hospital in southern Brazil. The outcome was patient-reported saliva-related quality of life, assessed using the saliva domain of the University of Washington Quality of Life questionnaire (UW-QOL); higher scores indicate better outcomes. Cognitive social capital was assessed using a brief three-item proxy index; participants were classified as scoring 3 (the maximum) or below 3. Hierarchical multiple linear regression estimated unstandardised coefficients (*B*) and 95% confidence intervals (95% CI), adjusting for education, tumour location, radiotherapy (yes vs. no), and time since treatment completion. Sensitivity analyses tested alternative operationalisations and adjustments.

**Results:**

A total of 105 adults participated (response rate 84.7%). The mean UW-QOL saliva-domain score was 61.85 ± 33.90, and 18 participants (17.1%) scored below 3. In the fully adjusted model, a score below 3 was associated with poorer saliva-related quality of life (*B* = −18.53; 95% CI −33.85 to −3.21; *p* = 0.018); sensitivity analyses were consistent.

**Conclusion:**

In this exploratory study, lower scores on a three-item cognitive social capital proxy index were associated with poorer saliva-related quality of life among head and neck cancer survivors. Perceived social resources may be relevant to this burden, but these preliminary findings require confirmation in larger longitudinal studies.

**Supplementary Information:**

The online version contains supplementary material available at https://doi.org/10.1007/s00520-026-11361-y.

## Introduction

Head and neck cancers comprise a heterogeneous group of malignancies arising in anatomically and functionally complex regions. Cancers of the lip and oral cavity, oropharynx, hypopharynx, and larynx account for approximately 771,000 new cases and 384,000 deaths worldwide [1]. Treatment commonly involves surgery, radiotherapy, systemic therapy, or combinations of these modalities, depending on tumour site, stage, and patient characteristics [2]. Despite advances in treatment planning and delivery, head and neck cancer treatment remains associated with clinically relevant acute and late adverse effects, including salivary dysfunction [3].

Salivary dysfunction is among the most frequent and persistent complications experienced by survivors, particularly after radiotherapy [4–6]. Damage to salivary gland tissue may result in reduced salivary secretion, changes in salivary composition, and persistent oral dryness, compromising oral function, dental health, and health-related quality of life [7, 8]. A systematic review of long-term oropharyngeal squamous cell carcinoma survivors identified persistent xerostomia, dysphagia, and dietary restrictions, even when global health-related quality of life was generally preserved [9]. These findings suggest that global scores may mask persistent domain-specific impairments and support the assessment of specific patient-reported outcomes, such as saliva-related quality of life.

The University of Washington Quality of Life questionnaire (UW-QOL) is a disease-specific instrument widely used in head and neck cancer research [10]. Its saliva domain captures perceived dry mouth problems and may help identify survivors requiring additional assessment and supportive care [11]. Thus, the saliva domain captures perceived dry mouth burden rather than objective salivary gland function or hyposalivation. Previous research has shown that saliva is frequently among the most impaired UW-QOL domains and among the problems most often selected as important by survivors [7].

Radiotherapy-related factors, tumour site, and sociodemographic characteristics have been associated with salivary symptoms and long-term health-related quality-of-life outcomes [6, 8, 9]. However, biological and treatment-related injury may not fully explain why individuals with similar clinical profiles report different levels of saliva-related impairment. The perceived burden of persistent symptoms may also be associated with psychosocial resources that help individuals understand their condition, obtain health information, mobilise assistance, access supportive care, and adapt to long-term limitations.

Social capital refers to resources inherent in and accessible through relationships and social networks [12]. Cognitive social capital reflects perceived trust, reciprocity, mutual support, and quality of social relationships [12, 13]. In chronic illness, these perceptions may shape help-seeking, information exchange, access to resources, and coping. Cognitive social capital is not expected to alter radiation-induced salivary gland damage; rather, it may be associated with how survivors experience, interpret, and manage its consequences in everyday life.

Higher levels of social capital have been associated with more favourable general and oral health outcomes, including better self-rated oral health, fewer oral impacts on daily activities, and better oral health-related quality of life [14, 15]. In a heterogeneous sample of cancer patients, greater social capital was associated with better quality of life [16]. In head and neck cancer, however, available research has predominantly examined social support rather than cognitive social capital. Prospective studies have found that greater social support before or during treatment was associated with better subsequent health-related quality of life [17, 18]. Although social support and cognitive social capital are related, they are conceptually distinct constructs. Evidence directly examining cognitive social capital in relation to specific functional outcomes among head and neck cancer survivors therefore remains scarce.

Understanding whether cognitive social capital is associated with saliva-related quality of life may broaden supportive-care perspectives beyond the biological consequences of cancer treatment. Such evidence may help identify survivors who could benefit from enhanced informational and psychosocial support, in addition to the clinical management of saliva-related problems. Therefore, this exploratory study aimed to evaluate the association between cognitive social capital and the UW-QOL saliva-domain score among head and neck cancer survivors. We hypothesised that lower cognitive social capital would be associated with poorer UW-QOL saliva-domain scores after accounting for relevant sociodemographic and clinical characteristics.

## Methods

### Ethical considerations

This study was reported in accordance with the Strengthening the Reporting of Observational Studies in Epidemiology (STROBE) statement [19]. The study was approved by the Human Research Ethics Committee of the Federal University of Santa Maria (CAAE 64691522.0.000.5346) and was conducted in accordance with the Declaration of Helsinki. All participants provided written informed consent.

### Study design, setting, eligibility criteria, and sample selection

This cross-sectional study included adults aged 18 years or older diagnosed with head and neck squamous cell carcinoma of the lip (C00), oral cavity (C02–C06), oropharynx (C01, C09–C10), hypopharynx (C12–C13), or larynx (C32), according to the International Classification of Diseases, 10th revision (ICD-10) [20]. Eligible participants had completed curative-intent treatment, including surgery, radiotherapy, chemotherapy, or combinations of these modalities, at least 3 months before data collection.

Participants were recruited at the Head and Neck Surgery outpatient clinic of the Hospital Universitário de Santa Maria, a public teaching hospital integrated into the Brazilian Unified Health System and located in the Centro-Oeste health macro-region of Rio Grande do Sul, southern Brazil. According to the State Oncology Plan, this macro-region comprises 44 municipalities and 1,000,225 inhabitants, based on the 2022 Brazilian Census [21]. Data collection was conducted from January 2023 to July 2025. During this period, consecutive eligible patients attending scheduled follow-up visits were invited to participate.

Sample size was estimated using G*Power 3.1.9.2 for the comparison of two independent means. Because no previous study was identified reporting effect sizes for the association between cognitive social capital and the UW-QOL saliva-domain score, the calculation was based on a previous study of head and neck cancer survivors that evaluated a conceptually related psychosocial exposure: participation in a support group [22]. The HNQOL eating domain was selected as the proxy outcome because it reflects oral functional limitations and includes dryness while eating, making it the closest available domain to saliva-related functional impairment among the outcomes reported in that study. The reported mean scores were 72.0 ± 20.0 among support-group participants and 52.2 ± 22.0 among non-participants, corresponding to a mean difference of 19.8 points and an estimated standardised effect size of 0.94. Support-group participation and cognitive social capital are not equivalent constructs, and the HNQOL eating domain and the UW-QOL saliva domain are not equivalent outcomes; this effect size was therefore used as a pragmatic planning estimate rather than as an expected effect size for the present association. Considering a two-sided alpha level of 5%, 90% power, and an assumed 1:1 ratio between exposure groups, the required sample size was 50 participants. After adding 10% to account for potential refusals or losses, the minimum required sample size was 55 participants. A sensitivity calculation based on the observed group sizes indicated that the study had 80% power to detect a standardised difference of approximately 0.73 and 90% power to detect a difference of approximately 0.85.

### Data collection and variables

Sociodemographic variables, including sex, age, self-reported skin colour, marital status, education, and household income, were obtained through structured face-to-face interviews. Data were collected by two postgraduate researchers trained to administer the sociodemographic questionnaire, the cognitive social capital questions, and the UW-QOL. Interviews were conducted in a private room at the Head and Neck Surgery outpatient clinic, using printed structured forms; questions were read exactly as written and the responses were recorded directly on the forms. The cognitive social capital and UW-QOL items used predefined response options, whereas the socioeconomic form also included numeric and free-text fields. Because data collection was fully structured, no qualitative interview guide was used. The instruments used are presented in Online Resource 1. Clinical variables, including tumour site, tumour stage, treatment modality, and time since treatment completion, were extracted from electronic medical records.

Tumour stage was recorded according to the 7th edition of the American Joint Committee on Cancer/Union for International Cancer Control TNM staging system [23] to ensure consistency across the study sample, because some participants had been staged before publication of the 8th edition.

### Quality of life assessment

Quality of life was assessed using the validated Brazilian Portuguese version of the University of Washington Quality of Life questionnaire (UW-QOL) [24]. The instrument includes 12 domains, each scored from 0 to 100: pain, appearance, activity, recreation, swallowing, chewing, speech, shoulder, taste, saliva, mood, and anxiety. Higher scores indicate better quality of life. In the present study, the outcome of interest was the UW-QOL saliva-domain score, which comprises four response options scored 100, 67, 33, and 0 (Online Resource 1).

### Cognitive social capital assessment

Cognitive social capital was measured using three individual-level proxy indicators previously used in oral health research [25, 26]. These indicators assessed perceived support in adverse situations, trust in friends and neighbours, and perceived good relationships in the neighbourhood. The three items were not administered as a shortened version of an existing instrument; they were selected a priori to capture complementary perception-based facets of cognitive social capital, using a brief format suitable for interviews with survivors attending routine follow-up visits.

The selected items are conceptually consistent with the cognitive dimension of social capital adopted in social capital measurement frameworks, including the Short Adapted Social Capital Assessment Tool (SASCAT) [27] and the Integrated Questionnaire for the Measurement of Social Capital (SC-IQ) [28], although the complete versions of these instruments were not applied. Items and response options are shown in Online Resource 1. Affirmative responses (Yes or Sometimes) were scored as 1. Negative responses (No) were scored as 0. The three items were summed to generate a cognitive social capital score ranging from 0 to 3, with higher scores indicating higher cognitive social capital. Responses of “Sometimes” were scored as 1 following the coding used in previous studies [25, 26]; an alternative coding, in which only “Yes” responses were scored, was tested in sensitivity analyses. This variable is therefore described as a three-item proxy index rather than a validated psychometric scale, and the distribution of the responses to each item and of the scores is presented in Online Resource 2 (Table S1).

### Statistical analysis

All analyses were performed using IBM SPSS Statistics, version 27.0 (IBM Corp., Armonk, NY, USA). Categorical variables were described using absolute and relative frequencies, and numerical variables using means, standard deviations, medians, and interquartile ranges.

The cognitive social capital score showed a marked ceiling effect (median = 3). For the primary analysis, participants were therefore classified as scoring 3 (i.e. answering “Yes” or “Sometimes” to all three items) or below 3 (i.e. answering “No” to at least one item). Age and time since treatment completion were dichotomised at the sample median for the bivariate comparisons; the dichotomised time variable was retained in the primary regression model, whereas age was not included in the multivariable models. The cut-off for time since treatment completion (26.2 months) corresponded to the sample median and was used pragmatically for the primary categorical analysis; it was not intended as a validated clinical threshold. The continuous variable was retained in a sensitivity analysis. Household income corresponded to the self-reported total family income and was dichotomised according to the Brazilian national minimum wage in force at the time of the interview (R$1302 from January to April 2023; R$1320 from May 2023; R$1412 in 2024; and R$1518 in 2025). Tumour site was grouped according to the expected proximity of the treatment field to salivary structures as lip/oral cavity/oropharynx and hypopharynx/larynx. Tumour stage was classified as initial (I/II) or advanced (III/IV).

Bivariate comparisons of UW-QOL saliva-domain scores according to sociodemographic, psychosocial, and clinical variables were performed using the Mann–Whitney *U* test, as the outcome was not normally distributed. Statistical significance was set at *p* < 0.05.

Hierarchical multiple linear regression, with sequential entry of blocks of variables, was used to estimate the association between cognitive social capital and the UW-QOL saliva-domain score. Although the saliva-domain score was not normally distributed, linear regression was used because its normality assumption refers to the distribution of the residuals rather than of the outcome and because it provides estimates as mean differences in UW-QOL points, which are directly interpretable; with more than 10 observations per variable, violations of this assumption are unlikely to affect confidence intervals and *p*-values substantially [29]. Unstandardised coefficients (*B*), 95% confidence intervals, and *p*-values were reported. Cognitive social capital was included as the main exposure in all models. The crude model included only cognitive social capital; Model 1 was adjusted for education; and Model 2 was additionally adjusted for tumour site, radiotherapy (yes vs. no), and time since treatment completion. Covariates were selected according to two criteria: an association with the outcome at *p* < 0.05 in the bivariate analysis and clinical plausibility as a determinant of saliva-related quality of life [6, 8, 9]. Given the sample size, the number of covariates was limited to preserve model parsimony. Tumour stage did not meet the statistical criterion and was not included in the main model; moreover, stage is expected to influence salivary outcomes mainly indirectly, through treatment decisions, particularly the indication and extent of radiotherapy, which was included in the model. A sensitivity analysis additionally adjusting for tumour stage was performed to assess whether this decision affected the main estimate.

Regression assumptions were assessed by visual inspection of residual plots, histograms, and normal probability plots. Standardised residuals were examined for potential outliers, and multicollinearity was evaluated using tolerance and variance inflation factor values.

Sensitivity analyses were performed to assess the robustness of the main estimate: (i) the original 0–3 cognitive social capital score entered as a numerical predictor; (ii) an alternative 0–3 score in which “Sometimes” responses were scored as 0 (i.e. only “Yes” responses were scored), also entered as a numerical predictor; (iii) the primary model additionally adjusted for tumour stage; (iv) the primary model with time since treatment completion entered as a continuous variable (natural log-transformed months); and (v) bias-corrected and accelerated (BCa) bootstrap 95% confidence intervals based on 1000 resamples. Because the score distributions differed across codings, standardised coefficients (*β*) were used to compare the magnitude of the associations.

## Results

A total of 124 eligible individuals were invited to participate, of whom 105 agreed, resulting in a response rate of 84.7%. Non-participation was attributed to inability to remain at the hospital for data collection because of dependence on public transportation.

The mean age of participants was 63.95 ± 8.50 years. Most were male (85.7%), white (74.3%), married, or living in a common-law relationship (56.2%) and had fewer than 8 years of formal education (69.5%). Most tumours were located in the lip/oral cavity or oropharynx (62.9%), 74.0% of participants with available staging data had advanced-stage disease, and 81.9% received radiotherapy. The mean time since completion of antineoplastic treatment was 41.9 ± 44.2 months, with a median of 26.2 months and a range from 3.0 to 192.0 months (1 month = 30.44 days). The observed median cognitive social capital score was 3: 87 participants (82.9%) obtained the maximum score, and 18 (17.1%) scored below 3 (score 2, *n* = 15; score 1, *n* = 2; score 0, *n* = 1) (Table 1; Online Resource 2, Table S1). Missing data occurred only for household income (*n* = 2), tumour stage (*n* = 1), and radiation dose (*n* = 12 among the 86 participants who received radiotherapy).

**Table 1:** Sociodemographic, psychosocial, and clinical characteristics of the sample and UW-QOL saliva-domain scores (*n* = 105)

Variables	*n* (%)	UW-QOL saliva-domain scores Mean ± SD	p*
Sex			
Female	15 (14.30)	53.20 ± 32.98	0.272
Male	90 (85.70)	63.28 ± 34.01	
Age^a^			
< 64 years	55 (52.40)	59.94 ± 31.81	0.497
≥ 64 years	50 (47.60)	63.94 ± 36.26	
Skin colour			
White	78 (74.30)	60.61 ± 35.63	0.587
Black or brown	27 (25.70)	65.40 ± 28.61	
Marital status			
Married/common-law relationship	59 (56.20)	61.50 ± 35.02	0.929
Single/divorced/widowed	46 (43.80)	62.28 ± 32.78	
Education level			
≥ 8 years of formal education	32 (30.50)	50.90 ± 32.89	**0.027**
< 8 years of formal education	73 (69.50)	66.64 ± 33.43	
Household income (Brazilian minimum wages)^b^			
> 1 Brazilian minimum wage	72 (69.90)	63.38 ± 34.20	0.436
≤ 1 Brazilian minimum wage	31 (30.10)	57.96 ± 33.42	
Cognitive social capital^a^			
Score 3	87 (82.90)	65.08 ± 32.63	**0.039**
Score < 3	18 (17.10)	46.22 ± 36.49	
Tumour location			
Lip/oral cavity or oropharynx	66 (62.90)	55.97 ± 33.79	**0.020**
Hypopharynx or larynx	39 (37.10)	71.79 ± 32.11	
Tumour stage^b^			
Initial (I and II)	27 (25.96)	70.33 ± 35.09	0.131
Advanced (III and IV)	77 (74.04)	59.25 ± 33.29	
Radiotherapy (yes vs. no)			
No	19 (18.10)	84.16 ± 28.16	**0.001**
Yes	86 (81.90)	56.92 ± 33.20	
Radiation dose (Gy)^a, b, c^			
≤ 66	42 (56.76)	58.64 ± 33.71	0.868
> 66	32 (43.24)	59.41 ± 33.68	
Time since treatment completion^a^			
> 26.2 months	52 (49.50)	71.77 ± 32.02	**0.003**
≤ 26.2 months	53 (50.50)	52.11 ± 33.13	

*UW-QOL* University of Washington Quality of Life Questionnaire, *SD* standard deviation, *Gy* gray

^*^Mann–Whitney *U* test

^a^Age, radiation dose, and time since treatment completion were dichotomised at the sample median; cognitive social capital was classified as the maximum score (3) versus a score below 3

^b^Missing data: household income (*n* = 2), tumour stage (*n* = 1), and radiation dose (*n* = 12 among participants who received radiotherapy)

^c^Radiation dose was analysed only among participants who received radiotherapy and had dose data available (*n* = 74)

Bold values indicate statistically significant results (*p* < 0.05)

The mean UW-QOL saliva-domain score was 61.85 ± 33.90, with a median of 67.00 (interquartile range: 33.00–100.00). Taste and saliva had the lowest mean scores among the UW-QOL domains, followed by chewing (Online Resource 2, Table S2).

In bivariate analyses, participants scoring below 3 had lower UW-QOL saliva-domain scores than those with the maximum score. Scores also differed according to education, tumour location, radiotherapy, and time since treatment completion. No statistically significant differences were observed for the remaining variables (Table 1).

In the unadjusted linear regression model, a cognitive social capital score below 3 was associated with an 18.86-point lower mean UW-QOL saliva-domain score compared with the maximum score (*B* = −18.86; 95% CI −35.96 to −1.76; *p* = 0.031). The model including only cognitive social capital explained 4.4% of the variance in saliva-domain scores (*R*^2^ = 0.044).

After adjustment for education, the association between a cognitive social capital score below 3 and the saliva-domain score remained similar in magnitude (*B* = −19.39; 95% CI −36.14 to −2.63; *p* = 0.024). The addition of education increased the explained variance to 9.3% (*R*^2^ = 0.093; adjusted *R*^2^ = 0.075), with a significant improvement in model fit (Δ*R*^2^ = 0.049; *p* = 0.021). In this model, fewer than 8 years of formal education were associated with a 16.16-point higher mean saliva-domain score compared with 8 or more years of education (95% CI 2.45 to 29.88; *p* = 0.021).

All 105 participants were included in the regression models, as there were no missing data for the variables included in the models. The subsequent addition of tumour location, radiotherapy and time since treatment completion explained a further 18.8% of the variance in saliva-domain scores (Δ*R*^2^ = 0.188; *p* < 0.001). The fully adjusted model was statistically significant (*F*[5,99] = 7.748; *p* < 0.001) and explained 28.1% of the variance in the outcome (*R*^2^ = 0.281; adjusted *R*^2^ = 0.245). A cognitive social capital score below 3 remained associated with a lower saliva-domain score after adjustment for education, tumour location, radiotherapy (yes vs. no), and time since treatment completion (*B* = −18.53; 95% CI −33.85 to −3.21; *p* = 0.018). Tumours located in the hypopharynx or larynx were associated with an 18.07-point higher mean score compared with tumours of the lip/oral cavity or oropharynx (95% CI 5.83 to 30.31; *p* = 0.004), whereas receipt of radiotherapy was associated with a 25.34-point lower mean score compared with no radiotherapy (95% CI −40.91 to −9.77; *p* = 0.002). Shorter time since treatment completion (≤ 26.2 months) was also associated with a 17.97-point lower mean saliva-domain score compared with longer time since treatment completion (95% CI −29.84 to −6.09; *p* = 0.003). Education was no longer associated with the saliva-domain score in the fully adjusted model (*B* = 3.95; 95% CI −9.33 to 17.23; *p* = 0.557) (Table 2).

**Table 2:** Hierarchical multiple linear regression analysis of the association between cognitive social capital and the UW-QOL saliva-domain score (*n* = 105)

Variable	*B* (95% CI)	*β*	*p*
***Unadjusted model***			
Cognitive social capital score < 3	−18.86 (−35.96 to −1.76)	−0.211	**0.031**
***Model 1***			
Cognitive social capital score < 3	−19.39 (−36.14 to −2.63)	−0.217	**0.024**
< 8 years of formal education	16.16 (2.45 to 29.88)	0.220	**0.021**
***Model 2***			
Cognitive social capital score < 3	−18.53 (−33.85 to −3.21)	−0.207	**0.018**
< 8 years of formal education	3.95 (−9.33 to 17.23)	0.054	0.557
Hypopharynx or larynx	18.07 (5.83 to 30.31)	0.259	**0.004**
Radiotherapy: yes	−25.34 (−40.91 to −9.77)	−0.289	**0.002**
Shorter time since treatment completion (≤ 26.2 months)	−17.97 (−29.84 to −6.09)	−0.266	**0.003**

Model 1 was adjusted for education. Model 2 was additionally adjusted for tumour location, radiotherapy, and time since treatment completion. Model fit: crude model, *R*^2^ = 0.044 and adjusted *R*^2^ = 0.035; Model 1, *R*^2^ = 0.093, adjusted *R*^2^ = 0.075, and Δ*R*^2^ = 0.049 (*p* = 0.021); Model 2, *R*^2^ = 0.281, adjusted *R*^2^ = 0.245, and Δ*R*^2^ = 0.188 (*p* < 0.001). Reference categories: cognitive social capital score of 3; ≥ 8 years of formal education; lip/oral cavity or oropharynx; no radiotherapy; longer time since treatment completion (> 26.2 months). Higher UW-QOL saliva-domain scores indicate better saliva-related quality of life. Statistically significant *p*-values are shown in bold

*B* unstandardised regression coefficient, *CI* confidence interval, *β* standardised regression coefficient, *UW-QOL* University of Washington Quality of Life Questionnaire

Diagnostic assessment of the final model indicated no relevant multicollinearity, with variance inflation factors ranging from 1.025 to 1.149. Standardised residuals ranged from −2.04 to 1.97, and visual inspection of the residual plots indicated an acceptable approximation to normality and no clear evidence of heteroscedasticity.

In sensitivity analyses, the direction of the association was consistent across all operationalisations of cognitive social capital. In the fully adjusted models, each one-point higher score was associated with a higher saliva-domain score using both the original coding (*B* = 12.36; 95% CI 0.95 to 23.77; *p* = 0.034) and the alternative coding (*B* = 7.70; 95% CI 0.99 to 14.41; *p* = 0.025), with standardised coefficients similar to that of the primary analysis (absolute *β* = 0.19 to 0.21). In the unadjusted models, these estimates were less precise and their confidence intervals included the null value. Additional adjustment for tumour stage (*n* = 104) did not materially change the association (*B* = −18.66; 95% CI −34.08 to −3.24; *p* = 0.018), and stage was not associated with the outcome (*B* = −2.47; 95% CI −16.57 to 11.62; *p* = 0.728). Modelling time since treatment completion as a continuous variable yielded similar results (*B* = −17.93; 95% CI −33.35 to −2.51; *p* = 0.023), as did bootstrap estimation (BCa 95% CI −34.70 to −2.26; *p* = 0.019). All sensitivity analyses are presented in Online Resource 2 (Table S3).

## Discussion

The main finding of this exploratory study was that lower scores on the three-item cognitive social capital proxy index were associated with poorer saliva-related quality of life in head and neck cancer survivors. This result is consistent with our conceptual hypothesis that survivors with lower perceived support, trust, and quality of social relationships would report a greater saliva-related burden. Salivary dysfunction and dry mouth remain among the most persistent toxicities of head and neck cancer treatment. The adjusted difference of approximately 19 points exceeds half the standard deviation of the saliva-domain score (approximately 17 points), a commonly used distribution-based benchmark for interpreting minimally important differences [30], and is also larger than the distribution-based minimal clinically important difference reported specifically for the UW-QOL saliva domain more than 3 months after treatment in a meta-analysis of patients with oral cavity and oropharyngeal cancer (15.00 points) [31]. It also corresponds to more than half of one response category of the saliva item. However, the confidence interval was wide, and its limit closest to the null (−3.2 points) is compatible with a small difference; the clinical relevance of this estimate should therefore be interpreted with caution.

In the prospective OraRad study, dry mouth and sticky saliva were among the oral health-related quality-of-life outcomes most persistently affected during the first 2 years following radiotherapy [8]. A recent multinational study showed that dry mouth and other late toxicities remained common among long-term head and neck cancer survivors and were associated with poorer oral health-related quality of life [32]. The association between shorter time since treatment completion and lower saliva-domain scores is consistent with previous evidence indicating that salivary outcomes vary over survivorship, although salivary dysfunction may persist [5]. Time since treatment may reflect partial recovery of salivary secretion, which has been documented objectively up to 2 years after radiotherapy, although salivary output often remains below pre-treatment levels [33, 34], as well as stabilisation of symptoms and adaptation to persistent treatment-related limitations. Previous studies have thus established the long-term clinical relevance of salivary symptoms; however, evidence directly examining the association between these symptoms and cognitive social capital in head and neck cancer survivors remains scarce. The present study extends existing evidence by suggesting that variation in a specific patient-reported functional outcome may be associated not only with tumour- and treatment-related characteristics but also with the perceived quality of the individual’s social environment.

Tumour site, stage, treatment, and time since treatment completion are interrelated. Site and stage influence the choice of treatment and the extent of the radiation field, which in turn affects the radiation dose to the major salivary glands, the main direct determinant of radiation-induced salivary gland damage. Time since treatment may reflect partial recovery of salivary secretion and adaptation to persistent symptoms, although the outcome assessed here is the perceived burden of dry mouth rather than salivary output. Within this framework, radiotherapy is the most proximal clinical determinant of saliva-related outcomes, whereas stage acts mainly upstream, through treatment choice.

In the education-adjusted model, fewer than 8 years of formal education were associated with higher saliva-domain scores; however, this association was no longer observed after adjustment for tumour location, radiotherapy and time since treatment completion, with the coefficient decreasing from 16.16 to 3.95. This pattern suggests confounding by clinical characteristics. In our sample, participants with fewer than 8 years of education more frequently had hypopharyngeal or laryngeal tumours (42.5% vs 25.0%; *p* = 0.088) and less frequently received radiotherapy (76.7% vs 93.8%; *p* = 0.037) than those with 8 or more years of education; both characteristics were associated with higher saliva-domain scores, possibly because these tumours are treated with radiation fields more distant from the major salivary glands, or without radiotherapy. Differences in expectations and response styles across educational levels and chance, given the small subgroups, may also have contributed.

Cognitive social capital refers to subjective and qualitative features of social relationships, such as trust, reciprocity, perceived support, and the perceived quality of neighbourhood relationships [12, 13]. This dimension may be particularly relevant to patient-reported outcomes because it reflects how individuals perceive the reliability and supportive capacity of their social environment. Previous studies in oral health and general quality of life have suggested that cognitive indicators of social capital may be more strongly associated with subjective health outcomes than structural indicators [26, 35]. Although these findings cannot be directly extrapolated to head and neck cancer survivors, they support the possibility that the perceived quality of social relationships may be relevant to saliva-related quality of life.

The study of social capital is particularly important in oncology because quality of life is a multidimensional construct influenced by physical symptoms, emotional responses, social functioning, access to resources, and the capacity to adapt to illness. Social capital may provide informational, emotional, and practical resources that are not captured by conventional clinical variables. In a heterogeneous sample of cancer patients, higher social capital was associated with better overall quality of life and better emotional and social functioning, as well as lower levels of nausea and vomiting, insomnia, appetite loss, and constipation [16]. Among patients with head and neck cancer, available evidence has focused mainly on social support rather than social capital. Prospective studies have shown that greater social support before or during treatment was associated with better subsequent health-related quality of life [17, 18]. Social support and cognitive social capital are not equivalent constructs; nevertheless, these findings collectively suggest that the perceived accessibility and reliability of social resources may be relevant to how patients experience cancer and its long-term consequences.

Several non-exclusive pathways may help explain the association observed in the present study. Trust, reciprocity, and perceived support may facilitate the exchange of health information, encourage patients to seek professional care, and increase their ability to mobilise assistance for appointments, transportation, self-care, and daily activities. These resources may also support adherence to symptom-management strategies, including hydration, dietary adaptation, oral hygiene, use of saliva substitutes or prescribed sialogogues when indicated, and regular dental follow-up. At the psychological level, confidence in supportive relationships may reduce feelings of isolation and provide emotional buffering against the stress generated by persistent symptoms. Trust and reciprocity have been proposed to influence health partly through the reduction of chronic stress, although the pathways remain incompletely understood [13]. Thus, cognitive social capital may be associated with how survivors appraise, cope with, and manage salivary symptoms rather than with biological saliva production itself. The association found in this study should therefore not be interpreted as evidence that cognitive social capital prevents or reverses radiation-induced glandular damage. The continued associations of radiotherapy, tumour location, and time since treatment completion with saliva-domain scores are consistent with the central role of biological, treatment-related, and survivorship-related determinants, while cognitive social capital may represent an additional psychosocial resource associated with the subjective burden of these effects.

Some limitations should be considered when interpreting the results. Because of the cross-sectional design, the direction of the association cannot be established. Lower scores on the cognitive social capital proxy index may be associated with poorer adaptation to salivary symptoms, but the reverse pathway is also plausible: persistent oral dryness and associated difficulties with eating, speaking, and sleeping may reduce social participation, weaken relationships, and negatively influence perceptions of available support. In addition, negative affectivity may simultaneously influence the perception of symptoms and of social resources. Longitudinal studies assessing social capital before treatment are needed to clarify the temporal sequence of this association. Although the final sample met the estimated sample size requirement, only 18 participants scored below 3, and the study had adequate power only to detect large differences (80% power for a standardised difference of approximately 0.73), which limited the precision of the adjusted estimates and may have led to overestimation of the magnitude of the association.

Cognitive social capital was assessed using a three-item proxy index rather than a comprehensive validated instrument, and this index has not been formally validated in head and neck cancer survivors. Although the selected items captured trust, neighbourhood relationships, and support during adverse situations, they may not encompass the full multidimensional nature of the construct, may overlap with perceived social support, and may not distinguish different forms of social capital. Because the score distribution was markedly skewed, the median-based cut-off effectively contrasted participants who answered “No” to at least one item with those who answered “Yes” or “Sometimes” to all three items; this dichotomisation discards information and may not capture meaningful gradients of cognitive social capital, although analyses using the original 0–3 score yielded associations in the same direction. Age and time since treatment completion were also dichotomised, which entails loss of information and may leave residual confounding.

In addition, both social capital and the saliva-domain score were obtained through self-report, raising the possibility of reporting bias. The fully adjusted model explained a limited proportion of the variance in saliva-domain scores (adjusted *R*^2^ = 0.245), indicating that relevant determinants were not measured. These include radiation dose to the parotid and submandibular glands, radiotherapy technique, concurrent chemotherapy, HPV status, use of xerogenic medications, comorbidities, tobacco and alcohol use, dental status, use of saliva substitutes or sialogogues, and objective salivary flow. Residual confounding by these factors, as well as by depressive symptoms or negative affectivity, which may influence both the perception of social resources and the reporting of symptoms, cannot be excluded. Recruitment from a single public healthcare service may limit generalisability to survivors receiving care in other healthcare systems or socioeconomic contexts, although the sample reflects the sociodemographic and clinical profile of many patients treated for head and neck cancer in publicly funded services. Strengths of the study include the use of a disease-specific and validated patient-reported outcome measure, adjustment for relevant sociodemographic and clinical factors, investigation of a specific functional outcome that is highly relevant in this population, and a set of sensitivity analyses supporting the robustness of the direction of the association.

These findings have potential implications for supportive care. Cognitive social capital may help identify survivors who experience both persistent oral symptoms and limited perceived social resources. Brief assessment of trust, perceived support, and availability of help during difficult situations could complement clinical assessment of saliva-related problems. Survivors reporting limited social resources might benefit from enhanced patient navigation, peer or support-group programmes, education on symptom management, referral to social and psychological services, and stronger coordination between oncology, dental, and community care. Such strategies should be culturally adapted and formally evaluated rather than assumed to be effective. Future multicentre and longitudinal studies should include larger samples, repeated measurements of social capital and saliva-related outcomes, comprehensive and validated measures of structural and cognitive social capital, and objective salivary assessments; the present exploratory study may provide preliminary data to inform such studies. They should also explore potential pathways involving coping, stress, adherence to symptom-management strategies, access to supportive care, and sense of coherence. In conclusion, in this exploratory study, lower scores on the three-item cognitive social capital proxy index were associated with poorer saliva-related quality of life among head and neck cancer survivors after adjustment for sociodemographic and clinical characteristics. These preliminary findings suggest that perceived social resources may be relevant to the patient-reported saliva-related burden and should be confirmed in larger longitudinal studies using comprehensive measures of social capital.

## Supplementary Information

Below is the link to the electronic supplementary material.

ESM 1 PDF (308 KB)

ESM 2 PDF (327 KB)

## Data Availability

The main data supporting the findings of this study are presented within the article and its Supplementary Information. Additional de-identified data may be made available from the corresponding author upon reasonable request.

## Declarations

### Use of AI-assisted technologies

During the preparation and revision of this manuscript, the authors used generative artificial intelligence tools (Claude, Anthropic; and ChatGPT, OpenAI) to assist with language editing, organisation, clarity, and refinement of academic writing. All AI-assisted content was critically reviewed and verified by the authors, who take full responsibility for the accuracy, integrity, interpretation, and final content of the manuscript.

## References

[1] Bray F, Laversanne M, Sung H et al (2024) Global cancer statistics 2022: GLOBOCAN estimates of incidence and mortality worldwide for 36 cancers in 185 countries. CA Cancer J Clin 74(3):229–263. 10.3322/caac.21834 38572751

[2] Caudell JJ, Gillison ML, Maghami E et al (2022) NCCN Guidelines® insights: head and neck cancers, version 1.2022. J Natl Compr Canc Netw 20(3):224–234. 10.6004/jnccn.2022.0016 35276673

[3] Ortigara GB, Bonzanini LIL, Schulz RE, Ferrazzo KL (2021) Late radiation effects in survivors of head and neck cancer: state of the science. Crit Rev Oncol Hematol 162:103335. 10.1016/j.critrevonc.2021.103335 33992801

[4] Jensen SB, Pedersen AM, Vissink A et al (2010) A systematic review of salivary gland hypofunction and xerostomia induced by cancer therapies: prevalence, severity and impact on quality of life. Support Care Cancer 18(8):1039–1060. 10.1007/s00520-010-0827-8 20237805

[5] Schulz RE, Bonzanini LIL, Ortigara GB et al (2021) Prevalence of hyposalivation and associated factors in survivors of head and neck cancer treated with radiotherapy. J Appl Oral Sci 29:e20200854. 10.1590/1678-7757-2020-0854 PMC8075291 33886946

[6] Abou-Bakr A, Hassanein FEA, William H, Ibrahim SS (2025) Prevalence of late xerostomia and hyposalivation with associated risk factors in survivors of head and neck cancer after radiotherapy: a multi-centric cross-sectional study. Radiat Oncol 20(1):162. 10.1186/s13014-025-02737-1 PMC12593948 41204325

[7] Bonzanini LIL, Soldera EB, Ortigara GB et al (2020) Clinical and sociodemographic factors that affect the quality of life of survivors of head and neck cancer. Support Care Cancer 28(4):1941–1950. 10.1007/s00520-019-05008-4 31375906

[8] Patton LL, Helgeson ES, Brennan MT et al (2023) Oral health-related quality of life after radiation therapy for head and neck cancer: the OraRad study. Support Care Cancer 31(5):286. 10.1007/s00520-023-07750-2 PMC11216743 37079106

[9] Zarrella D, Chitnis O, Kim HE et al (2026) Long-term health-related quality of life in oropharyngeal squamous cell carcinoma: a systematic review. Otolaryngol Head Neck Surg 174(6):1421–1433. 10.1002/ohn.70219 41886789

[10] Laraway DC, Rogers SN (2012) A structured review of journal articles reporting outcomes using the University of Washington Quality of Life Scale. Br J Oral Maxillofac Surg 50(2):122–131. 10.1016/j.bjoms.2010.12.005 21239091

[11] Rogers SN, Johnson IA, Lowe D (2010) Xerostomia after treatment for oral and oropharyngeal cancer using the University of Washington saliva domain and a xerostomia-related quality-of-life scale. Int J Radiat Oncol Biol Phys 77(1):16–23. 10.1016/j.ijrobp.2009.04.021 19679404

[12] Moore S, Kawachi I (2017) Twenty years of social capital and health research: a glossary. J Epidemiol Community Health 71(5):513–517. 10.1136/jech-2016-208313 28087811

[13] Abbott S, Freeth D (2008) Social capital and health: starting to make sense of the role of generalized trust and reciprocity. J Health Psychol 13(7):874–883. 10.1177/1359105308095060 18809638

[14] Rouxel P, Tsakos G, Demakakos P, Zaninotto P, Watt RG (2015) Social capital and oral health among adults 50 years and older: results from the English Longitudinal Study of Ageing. Psychosom Med 77(8):927–937. 10.1097/PSY.0000000000000197 26458157

[15] Knorst JK, Vettore MV, Ardenghi TM (2022) Social capital and oral health promotion: past, present, and future challenges. Front Oral Health 3:1075576. 10.3389/froh.2022.1075576 PMC9732376 36507311

[16] Pitis A, Diamantopoulou M, Panagiotou A, Papageorgiou D, Tzavella F (2025) Social capital as a determinant of quality of life in Greek cancer patients: a cross-sectional study. Front Public Health 13:1602804. 10.3389/fpubh.2025.1602804 PMC12394470 40893209

[17] Karnell LH, Christensen AJ, Rosenthal EL, Magnuson JS, Funk GF (2007) Influence of social support on health-related quality of life outcomes in head and neck cancer. Head Neck 29(2):143–146. 10.1002/hed.20501 17111431

[18] Howren MB, Christensen AJ, Hynds Karnell L, Van Liew JR, Funk GF (2013) Influence of pretreatment social support on health-related quality of life in head and neck cancer survivors: results from a prospective study. Head Neck 35(6):779–787. 10.1002/hed.23029 22715128

[19] Von Elm E, Altman DG, Egger M et al (2007) The Strengthening the Reporting of Observational Studies in Epidemiology (STROBE) statement: guidelines for reporting observational studies. Lancet 370(9596):1453–1457. 10.1016/S0140-6736(07)61602-X 18064739

[20] World Health Organization (2010) International statistical classification of diseases and related health problems. 10th revision, vol 2. https://icd.who.int/browse10/Content/statichtml/ICD10Volume2_en_2010.pdf. Accessed 10 February 2026

[21] Rio Grande do Sul, Secretaria da Saúde (2024) Plano de ação estadual de oncologia: triênio 2024–2026, 5ª ed. https://saude.rs.gov.br/upload/arquivos/202407/17150639-plano-estadual-de-oncologia-2024-2026.pdf. Accessed 19 July 2026

[22] Vakharia KT, Ali MJ, Wang SJ (2007) Quality-of-life impact of participation in a head and neck cancer support group. Otolaryngol Head Neck Surg 136(3):405–410. 10.1016/j.otohns.2006.10.018 17321868

[23] Edge SB, Byrd DR, Compton CC (eds) (2010) AJCC cancer staging manual, 7th edn. Springer, New York

[24] Vartanian JG, Carvalho AL, Yueh B et al (2006) Brazilian-Portuguese validation of the University of Washington Quality of Life Questionnaire for patients with head and neck cancer. Head Neck 28(12):1115–1121. 10.1002/hed.20464 16823873

[25] Knorst JK, Menegazzo GR, Emmanuelli B, Mendes FM, Ardenghi TM (2019) Effect of neighborhood and individual social capital in early childhood on oral health-related quality of life: a 7-year cohort study. Qual Life Res 28(7):1773–1782. 10.1007/s11136-019-02138-4 30963360

[26] Knorst JK, Vettore MV, Brondani B, Emmanuelli B, Ardenghi TM (2023) The different roles of structural and cognitive social capital on oral health-related quality of life among adolescents. Int J Environ Res Public Health 20(8):5603. 10.3390/ijerph20085603 PMC10138599 37107885

[27] De Silva MJ, Harpham T, Tuan T, Bartolini R, Penny ME, Huttly SR (2006) Psychometric and cognitive validation of a social capital measurement tool in Peru and Vietnam. Soc Sci Med 62(4):941–953. 10.1016/j.socscimed.2005.06.050 16095787

[28] Grootaert C, Narayan D, Nyhan Jones V, Woolcock M (2004) Measuring social capital: an integrated questionnaire. World Bank Working Paper No. 18. World Bank, Washington, DC. 10.1596/0-8213-5661-5

[29] Schmidt AF, Finan C (2018) Linear regression and the normality assumption. J Clin Epidemiol 98:146–151. 10.1016/j.jclinepi.2017.12.006 29258908

[30] Norman GR, Sloan JA, Wyrwich KW (2003) Interpretation of changes in health-related quality of life: the remarkable universality of half a standard deviation. Med Care 41(5):582–592. 10.1097/01.MLR.0000062554.74615.4C 12719681

[31] Binenbaum Y, Amit M, Billan S, Cohen JT, Gil Z (2014) Minimal clinically important differences in quality of life scores of oral cavity and oropharynx cancer patients. Ann Surg Oncol 21(8):2773–2781. 10.1245/s10434-014-3656-z 24710774

[32] Westgaard KL, Amdal CD, Taylor KJ et al (2025) Oral health-related quality of life among long-term head and neck cancer survivors: a multinational study. Support Care Cancer 33(10):868. 10.1007/s00520-025-09930-8 PMC12450221 40975748

[33] Li Y, Taylor JMG, Ten Haken RK, Eisbruch A (2007) The impact of dose on parotid salivary recovery in head and neck cancer patients treated with radiation therapy. Int J Radiat Oncol Biol Phys 67(3):660–669. 10.1016/j.ijrobp.2006.09.021 PMC2001308 17141973

[34] Lin A, Helgeson ES, Treister NS et al (2022) The impact of head and neck radiotherapy on salivary flow and quality of life: results of the ORARAD study. Oral Oncol 127:105783. 10.1016/j.oraloncology.2022.105783 PMC8977268 35231809

[35] Buja A, Akhtar S (2025) The association between social capital and quality of life in old adults: a systematic review and meta-analysis. Front Public Health 13:1668696. 10.3389/fpubh.2025.1668696 PMC12629930 41283054

