# Supplementary material for: Association between cognitive social capital and saliva-related quality of life among head and neck cancer survivors: a cross-sectional study
Source: Support Care Cancer. 2026 Oct 22;34(11):1141. doi: 10.1007/s00520-026-11361-y (PMC13690865; doi:10.1007/s00520-026-11361-y)
Supplement: Supplementary file 1 [file 520_2026_11361_MOESM1_ESM.pdf]

Supplementary Materials

Online Resource 1

Article title: Association between cognitive social capital and saliva-related quality of life among head and neck cancer survivors: a cross-sectional study

Journal: Supportive Care in Cancer

Authors: Alan Carvalho Daros; Jessica Klöckner Knorst; Bruno Emmanuelli; João Alberto de Almeida Gonçalves; Raquel Pippi Antoniazzi; Kívia Linhares Ferrazzo

Corresponding author: Kívia Linhares Ferrazzo, Department of Pathology, Federal University of Santa Maria, Santa Maria, Rio Grande do Sul, Brazil. E-mail: kivialinhares@uol.com.br

Study instruments and definition of variables

All instruments were administered in Brazilian Portuguese during face-to-face structured interviews conducted by trained interviewers. The English versions presented below were translated by the authors, except for the UW-QOL item, which is shown in its validated Brazilian Portuguese version and original English wording.

1. Structured interview: socioeconomic characterisation

Item	Question	Response format
1	Are you literate?	( ) Yes ( ) No
2	What is your occupation?	Open answer
3	How many years of formal education have you completed?	____ years
4	How many people, including yourself, live in your household?	____ people
5	How many of them are over 35 years of age?	____ people
6	How many rooms are there in your home (excluding bathrooms)?	____ rooms
7	Family structure: whom do you live with?	( ) Spouse/partner<br>( ) Spouse/partner and/or children<br>( ) Alone<br>( ) Other
8	Last month, how much did all the people who live in your household receive together, in Brazilian reais?	R$ ____

Items 3 (education) and 8 (household income) were used in the analyses; their categorisation is described in Section 4. Sex, age, self-reported skin colour and marital status were also recorded during the interview.

2. Cognitive social capital (three-item proxy index)

Question	Response options	Score
1. If something unfortunate happened to you, would someone help you in this situation?	( ) Yes<br>( ) No	Yes = 1<br>No = 0

Question	Response options	Score
2. Can your neighbours and friends be trusted?	( ) Yes<br>( ) Sometimes<br>( ) No	Yes or Sometimes = 1<br>No = 0
3. Do most people in your neighbourhood generally have good relationships with each other?	( ) Yes<br>( ) Sometimes<br>( ) No	Yes or Sometimes = 1<br>No = 0

The items were selected as proxy indicators of cognitive social capital previously used in oral-health research (Knorst et al., 2019; Knorst et al., 2023) and are conceptually consistent with the cognitive dimension of social capital described in the SASCAT (De Silva et al., 2006) and the SC-IQ (Grootaert et al., 2004). Responses "Yes" or "Sometimes" were assigned 1 point and "No" was assigned 0 points; the item scores were summed (range 0–3). In the primary analysis, participants were classified as scoring 3 (answering "Yes" or "Sometimes" to all three items) or below 3 (answering "No" to at least one item). In a sensitivity analysis, an alternative coding was used in which "Sometimes" responses were scored as 0 (i.e., only "Yes" responses were scored).

3. University of Washington Quality of Life Questionnaire (UW-QOL v4): saliva domain

Saliva (check one box) / Saliva (marque uma alternativa), referring to the past 7 days:

Brazilian Portuguese version (as administered)	Original English version	Score
( ) Minha saliva é de consistência normal	( ) My saliva is of normal consistency.	100
( ) Eu tenho menos saliva que o normal, mas ainda é o suficiente	( ) I have less saliva than normal, but it is enough.	67
( ) Eu tenho muito pouca saliva	( ) I have too little saliva.	33
( ) Eu não tenho saliva	( ) I have no saliva.	0

The validated Brazilian Portuguese version of the UW-QOL was administered; wording and scores are shown as published in its validation study (Vartanian et al., 2006). The complete UW-QOL v4 questionnaire (© University of Washington) is publicly available and was applied in full; only the saliva domain was used as the study outcome.

4. Definition and categorisation of study variables

Variable	Source	Information collected	Categorisation used in the analyses
Sex	Interview	Sex	Male; female
Age	Interview	Age in years	< 64 years; ≥ 64 years (sample median)
Skin colour	Interview	Self-reported skin colour	White; Black or brown
Marital status	Interview	Marital status	Married or common-law relationship; single, divorced or widowed
Education	Interview (Section 1, item 3)	Years of formal education	≥ 8 years; < 8 years

Variable	Source	Information collected	Categorisation used in the analyses
Household income	Interview (Section 1, item 8)	Self-reported total family income in the previous month	> 1 national minimum wage; ≤ 1 national minimum wage<br>Minimum wage in force at the time of the interview: R$1,302 (January to April 2023); R$1,320 (from May 2023); R$1,412 (2024); R$1,518 (2025)
Cognitive social capital	Interview (Section 2)	Three-item proxy index (score 0–3)	Primary analysis: score 3; score < 3. Sensitivity analyses: 0–3 score (original coding and alternative coding with "Sometimes" = 0)
Tumour location	Medical records	Primary tumour site (ICD-10)	Lip, oral cavity or oropharynx; hypopharynx or larynx
Tumour stage	Medical records	TNM stage (AJCC/UICC, 7th edition)	Initial (I and II); advanced (III and IV)
Radiotherapy	Medical records	Receipt of radiotherapy	Yes; no
Radiation dose	Medical records	Total radiation dose (Gy)	≤ 66 Gy; > 66 Gy (sample median)
Time since treatment completion	Medical records	Time since completion of antineoplastic treatment, in months (1 month = 30.44 days)	Primary analysis: ≤ 26.2 months; > 26.2 months (sample median). Sensitivity analysis: natural log-transformed months
Saliva-related quality of life (outcome)	Interview (Section 3)	UW-QOL saliva-domain score	Score from 0 to 100 (higher scores indicate better saliva-related quality of life)

References

De Silva MJ, Harpham T, Tuan T, Bartolini R, Penny ME, Huttly SR (2006) Psychometric and cognitive validation of a social capital measurement tool in Peru and Vietnam. Soc Sci Med 62(4):941–953. https://doi.org/10.1016/j.socscimed.2005.06.050

Grootaert C, Narayan D, Nyhan Jones V, Woolcock M (2004) Measuring social capital: an integrated questionnaire. World Bank Working Paper No. 18. World Bank, Washington, DC. https://doi.org/10.1596/0-8213-5661-5

Knorst JK, Menegazzo GR, Emmanuelli B, Mendes FM, Ardenghi TM (2019) Effect of neighborhood and individual social capital in early childhood on oral health-related quality of life: a 7-year cohort study. Qual Life Res 28(7):1773–1782. https://doi.org/10.1007/s11136-019-02138-4

Knorst JK, Vettore MV, Brondani B, Emmanuelli B, Ardenghi TM (2023) The different roles of structural and cognitive social capital on oral health-related quality of life among adolescents. Int J Environ Res Public Health 20(8):5603. https://doi.org/10.3390/ijerph20085603

Vartanian JG, Carvalho AL, Yueh B et al (2006) Brazilian-Portuguese validation of the University of Washington Quality of Life Questionnaire for patients with head and neck cancer. Head Neck 28(12):1115–1121. https://doi.org/10.1002/hed.20464
