# Supplementary material for: Association between cognitive social capital and saliva-related quality of life among head and neck cancer survivors: a cross-sectional study
Source: Support Care Cancer. 2026 Oct 22;34(11):1141. doi: 10.1007/s00520-026-11361-y (PMC13690865; doi:10.1007/s00520-026-11361-y)
Supplement: Supplementary file 2 [file 520_2026_11361_MOESM2_ESM.pdf]

Supplementary Materials

Online Resource 2

Article title: Association between cognitive social capital and saliva-related quality of life among head and neck cancer survivors: a cross-sectional study

Journal: Supportive Care in Cancer

Authors: Alan Carvalho Daros; Jessica Klöckner Knorst; Bruno Emmanuelli; João Alberto de Almeida Gonçalves; Raquel Pippi Antoniazzi; Kívia Linhares Ferrazzo

Corresponding author: Kívia Linhares Ferrazzo, Department of Pathology, Federal University of Santa Maria, Santa Maria, Rio Grande do Sul, Brazil. E-mail: kivialinhares@uol.com.br

Supplementary Table S1. Distribution of responses to the cognitive social capital items and of the cognitive social capital score (n = 105)

Item	Yes, n (%)	Sometimes, n (%)	No, n (%)
1. If something unfortunate happened to you, would someone help you in this situation?	98 (93.3)	—	7 (6.7)
2. Can your neighbours and friends be trusted?	61 (58.1)	35 (33.3)	9 (8.6)
3. Do most people in your neighbourhood generally have good relationships with each other?	77 (73.3)	22 (21.0)	6 (5.7)

Cognitive social capital score	Original coding, n (%)	Alternative coding (“Sometimes” = 0), n (%)
3	87 (82.9)	51 (48.6)
2	15 (14.3)	33 (31.4)
1	2 (1.9)	17 (16.2)
0	1 (1.0)	4 (3.8)
Median	3	2

Original coding: “Yes” or “Sometimes” = 1 point and “No” = 0 points. Alternative coding (sensitivity analysis): only “Yes” = 1 point; “Sometimes” or “No” = 0 points. —: response option not available for this item.

Supplementary Table S2. University of Washington Quality of Life Questionnaire (UW-QOL) domain scores (n = 105)

UW-QOL domain	Mean ± SD	Median (IQR)
Pain	75.95 ± 26.84	75.00 (50.00–100.00)
Appearance	92.86 ± 14.99	100.00 (100.00–100.00)
Activity	80.48 ± 21.92	75.00 (75.00–100.00)
Recreation	82.86 ± 25.78	100.00 (75.00–100.00)
Swallowing	69.89 ± 33.87	67.00 (33.00–100.00)
Chewing	64.76 ± 33.20	50.00 (50.00–100.00)
Speech	76.01 ± 25.08	67.00 (67.00–100.00)
Shoulder	76.50 ± 31.40	100.00 (67.00–100.00)
Taste	60.61 ± 38.41	67.00 (33.00–100.00)
Saliva	61.85 ± 33.90	67.00 (33.00–100.00)
Mood	75.95 ± 29.20	75.00 (50.00–100.00)
Anxiety	78.42 ± 35.52	100.00 (67.00–100.00)

Scores range from 0 (worst) to 100 (best). SD: standard deviation; IQR: interquartile range (25th to 75th percentile).

Supplementary Table S3. Primary and sensitivity analyses of the association between cognitive social capital and the UW-QOL saliva-domain score

Analysis	n	Unadjusted model, B (95% CI)	Model 1, B (95% CI)	Fully adjusted model, B (95% CI)	β (fully adjusted)	R^2 (fully adjusted)
**Primary analysis**						
Score < 3 vs 3 (reference)	105	−18.86 (−35.96 to −1.76) p = 0.031	−19.39 (−36.14 to −2.63) p = 0.024	−18.53 (−33.85 to −3.21) p = 0.018	−0.207	0.281
**Sensitivity analyses**						
(i) 0–3 score, original coding (per one-point increase)	105	12.00 (−0.70 to 24.69) p = 0.064	13.04 (0.60 to 25.48) p = 0.040	12.36 (0.95 to 23.77) p = 0.034	0.187	0.273
(ii) 0–3 score, alternative coding with “Sometimes” = 0 (per one-point increase)	105	5.49 (−2.10 to 13.09) p = 0.155	5.51 (−1.94 to 12.96) p = 0.146	7.70 (0.99 to 14.41) p = 0.025	0.196	0.277
(iii-a) Additional adjustment for tumour stage: score < 3 vs 3	104	—	—	−18.66 (−34.08 to −3.24) p = 0.018	−0.209	0.286
(iii-b) Additional adjustment for tumour stage: 0–3 score, original coding	104	—	—	12.44 (0.95 to 23.94) p = 0.034	0.189	0.277
(iv) Time since treatment completion as a continuous variable (natural log-transformed months): score < 3 vs 3	105	—	—	−17.93 (−33.35 to −2.51) p = 0.023	−0.200	0.269
(v) Bias-corrected and accelerated bootstrap 95% CI (1,000 resamples): score < 3 vs 3	105	−18.86 (−36.44 to −1.21) p = 0.033	−19.39 (−36.14 to −1.97) p = 0.031	−18.53 (−34.70 to −2.26) p = 0.019	—	—

B: unstandardised regression coefficient; CI: confidence interval; β: standardised regression coefficient; R^2: coefficient of determination. Model 1: adjusted for education. Fully adjusted model: adjusted for education, tumour location, radiotherapy and time since treatment completion (≤ 26.2 vs > 26.2 months); in analyses (iii-a) and (iii-b), additionally adjusted for tumour stage (I–II vs III–IV; one participant with missing staging data was excluded); in analysis (iv), time since treatment completion was entered as natural log-transformed months instead of the dichotomised variable. In the primary analysis and in analyses (iii-a), (iv) and (v), negative coefficients indicate lower saliva-domain scores among participants with a score below 3. In analyses (i), (ii) and (iii-b), positive coefficients indicate higher saliva-domain scores for each one-point higher cognitive social capital score. In analysis (iv), natural log-transformed time since treatment completion was positively associated with the saliva-domain score (B = 6.82; 95% CI 1.79 to 11.85; p = 0.008), corresponding to approximately 4.73 points per doubling of time. Original coding: “Yes” or “Sometimes” = 1 point; alternative coding: “Sometimes” = 0 points. In analysis (v), p-values were derived from the bootstrap procedure. —: not estimated.
